# A facile approach for the determination of degree of deacetylation of chitosan using acid-base titration

**DOI:** 10.1016/j.heliyon.2022.e09924

**Published:** 2022-07-11

**Authors:** Joydeep Dutta, Priyanka

**Affiliations:** Department of Chemistry, Amity School of Applied Sciences, Amity University Haryana, Gurgaon 122413, Haryana, India

**Keywords:** Degree of deacetylation, Chitosan, Acid-base titration, New equation, Ease of calculation, ^13^C-NMR

## Abstract

Several spectroscopic techniques such as nuclear magnetic resonance (NMR), UV-visible, Fourier transform infrared (FT-IR), etc. have been already used for the determination of degree of deacetylation (DD) of chitosan. These techniques involve the interpretation of spectral data apart from sample preparation for obtaining DD of chitosan. In addition, inaccurate interpretation of data sometimes misleads researchers to get an exact value of DD of chitosan. Among them, NMR is an excellent technique for the estimation of DD of chitosan but expensive and not found easily in every research laboratory. On the other hand, titrimetric methods have been employed by many researchers for determining the DD of chitosan but these existing methods involve many complex calculations, which do not always give accurate results. Moreover, few of the acid-base titration methods are little complicated for execution. Therefore, in this present study, we adopted a very handy and simple acid-base titration method with a new approach and proposed a new equation facilitating the ease of calculation that is not reported elsewhere for the determination of DD value by observing the net volume of NaOH consumed for the complete neutralization of protonated amino groups (-NH_3_^+^) of chitosan describing the novelty of the work. All the DD values (77.04 ± 1.36; 81.71 ± 1.73; 91.68 ± 1.42 for CS1, CS2, and CS3 respectively) obtained for various chitosan samples were in good agreement with the reported DD values (>75%, >80%, and >85% for CS1, CS2, and CS3 respectively) mentioned in the specifications of chitosan samples supplied by the manufacturer. Finally, the experimental DD values were further validated with the DD values (77.39%, 81.64%, and 90.5% for CS1, CS2, and CS3 respectively) obtained from the interpretation of ^13^C-NMR spectral data and all the experimental DD values were consistent with the DD values as calculated based on NMR spectra.

## Highlights

• The acid-base titration method with a new approach reported in this article for the determination of degree of deacetylation of chitosan provides an accuracy, reproducibility, and reliability.
• In addition, the reported method with a new approach is very convenient as compared to other existing methods to determine the degree of deacetylation of chitosan.
• The newly introduced equation to estimate degree of deacetylation of chitosan is very simple and convenient.
• The DD values of chitosan obtained by the acid-base titration method are perfectly validated based on ^13^C-NMR data.

## Graphical abstract

**Figure:**
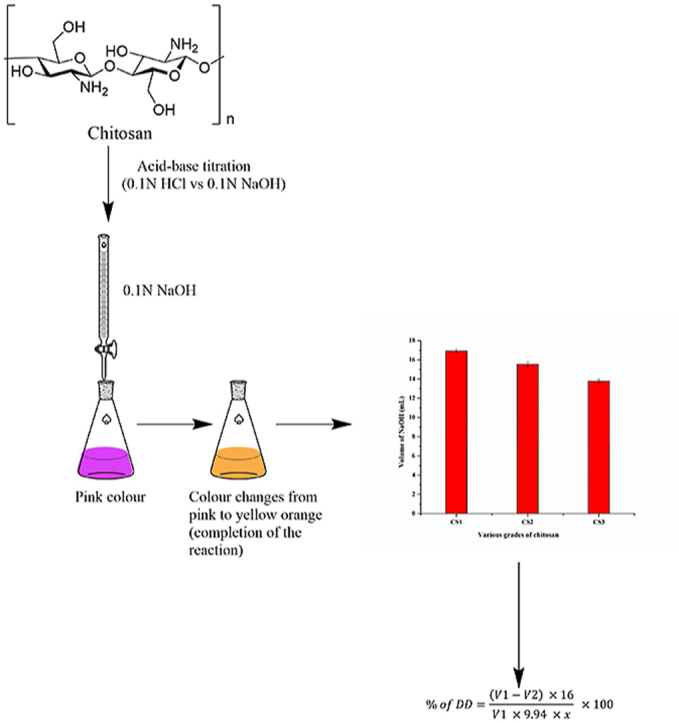

## 1 Introduction

Chitosan is a copolymer of N-acetyl-D-glucosamine and D-glucosamine units and it is derived from chitin, which is the second most abundant natural polysaccharide next to cellulose [1]. It is mainly extracted from the exoskeletons of insects, crustaceans, shrimp shells, crabs, lobsters, the cell walls of fungi, and algae to name a few through partial deacetylation of chitin [2, 3, 4]. Owing to its outstanding and remarkable physicochemical characteristics including biodegradability, biocompatibility, non-toxicity, and antimicrobial properties, still the researchers have been interestingly using chitosan for various purposes such as medicine, tissue engineering, drug delivery, food packaging, environmental protection, and cosmetics [5, 6, 7, 8, 9]. However, the DD of chitosan is an important factor that not only defines the molar fraction of deacetylated units in its polymeric chain but also greatly affects its own solubility, viscosity, ion-exchange capacity, flocculation ability, and amino group reaction [10]. Increasing the degree of deacetylation of chitosan to a certain extent facilitates its solubility, which in turn, is also responsible for influencing its antimicrobial characteristics [11]. In addition, other biological properties namely analgesic, antioxidant, haemostatic, and mucoadhesive abilities increase with increasing its degree of deacetylation [11, 12]. Therefore, it is mandatory to know the DD value of chitosan, which is to be taken into consideration for carrying out any sort of experimental work for research purposes. In order to determine the DD value of chitosan, various researchers have reported the use of different methods such as pH-metric titration, UV-vis spectroscopy, infrared spectroscopy, elemental analysis, ^1^H and ^13^C NMR spectroscopy to name a few but due to their corresponding shortcomings in terms of determining the DD of chitosan have rendered them to explore very simple technique [13, 14]. Notably, NMR is an excellent technique for the determination of DD of chitosan. Only the cost of this instrument as well as the chemicals required for sample preparation has restricted its uses. Moreover, the acid-base titration and potentiometric titration appear to be more appropriate in this context and cost-effective for determining the DD of chitosan [15, 16]. Yao *et al.* [17] demonstrated the determination of DD of chitooligosaccharides (COS) of different molecular weights with the help of the Chinese Pharmacopoeia’s acid-base titration method using methyl orange as an indicator. In another study, Hossain and Uddin [18] also reported the determination of DD of chitosan by acid-base titration method using methyl orange as an indicator. For both the cases, they separately used different equations for calculating DD value of chitosan. Similarly, Varan [19] also reported the use of acid-base titration method for the determination of DD of chitosan using an entirely different equation. In another attempt, Youling *et al.* [20] also used acid-base titration method to determine DD value of chitosan. Still there are some loopholes in the equations as well as in the methods and these are not explicitly addressed by none of them. In addition, some of the reported acid-base titration methods are little tricky.

Therefore, in this present study, we adopted the acid-base titration method [1] with a new approach rather in a simplified way apart from using a new equation to determine DD value of our chitosan samples, which is expected to provide an accuracy, reproducibility, and reliability. To validate the data obtained by this method, ^13^C-NMR spectroscopy was successfully employed. Further, to the best of our knowledge, the proposed equation is not reported elsewhere yet and it would be the robust equation over the existing equations and could be successfully used for the estimation of DD value of chitosan via acid-base titration.

## 2 Experimental

### 2.1 Materials

Three different grades of chitosan (CS) having degree of deacetylations >75% (viscosity = 1040 mPa.s), >80% (viscosity = 372 mPa.s), and >85% (viscosity = 618 mPa.s) further assigned as CS1, CS2 and CS3 respectively were purchased from the Marine Hydrocolloids, Kochi, Kerala, India. Hydrochloric acid (HCl) was supplied by Fisher Scientific India Pvt Ltd., Powai, Mumbai. Sodium hydroxide pellets purified (NaOH) and methyl orange pH indicator were purchased from Central Drug House (P) Ltd. Corp., Daryaganj, New Delhi. All the chemicals including chitosan samples were used as received without further purification.

### 2.2 Preparation of chitosan solution

Chitosan solution was prepared with modification according to the method reported by Hossain and Uddin [18]. Briefly, 1g of each grade of chitosan samples namely CS1, CS2, and CS3 was dried in a hot air oven at 110 °C for 1h. After drying and further weighing, the said chitosan samples were individually added into a freshly prepared 100 mL of 0.1 N HCl and stirred vigorously for 2h at room temperature for complete dissolution. Notably, the freshly prepared chitosan solution made with the dried chitosan for each case was used as a stock solution for completing the whole titration process.

### 2.3 Acid-base titration method

**Figure 1:**
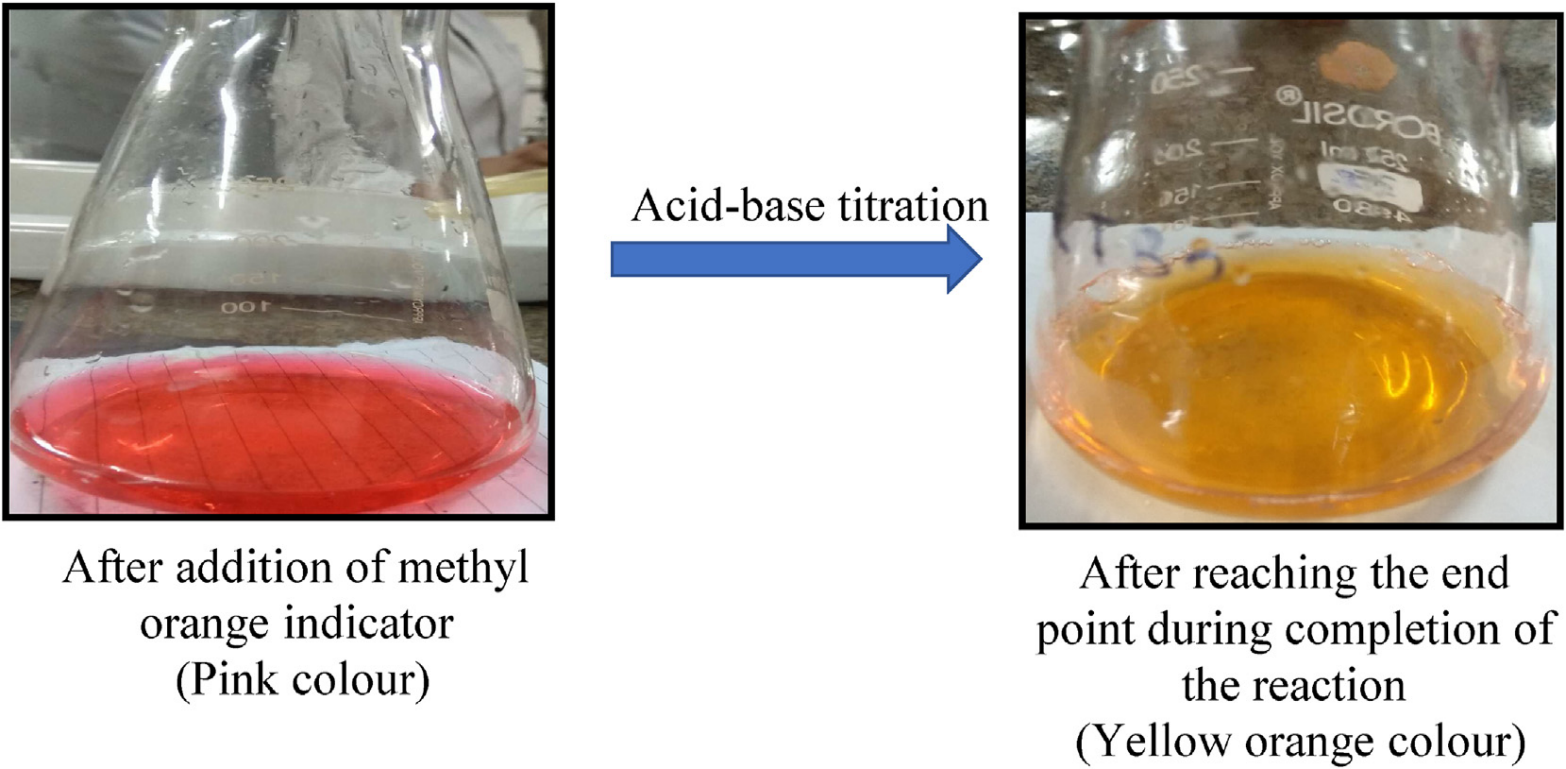
Prompt visualization of colour transition from pink to yellow orange during acid-base titration.

Acid-base titration was adopted to determine degree of deacetylation of chitosan. Succinctly, 100 mL of 0.1 N NaOH solution was freshly prepared every time as a standard solution for titration against each chitosan sample. After that an aliquot of each chitosan solution was separately transferred into a conical flask and titrated against the standard NaOH solution. It is worth mentioning that every time same volume of chitosan solution should be used for carrying out titration. In our case, we used 30 mL of chitosan solution for each titration, but it may vary depending on the experimental design done by individual users. Then, into each conical flask containing several grades of chitosan solution, 2–3 drops of methyl orange indicator were added and titrated against the standard NaOH solution until end point reached. The end point was detected by visual inspection through a sharp colour change from pink to yellow orange. For prompt and comprehensive visualization, the colour transition is shown in Figure 1. Finally, the volume of NaOH solution consumed was noted down. The same experiment was performed thrice against each chitosan sample. The DD of each chitosan sample was calculated using the following equation as established by the authors:

(1) $$\%\;of\;DD=\frac{\left(V1-V2\right)\;\times 16}{V1\;\times 9.94\;\times x}\;\times 100$$

where, x is the weight of dried chitosan; V1 = volume of chitosan solution prepared in 0.1 N HCl solution in mL; V2 = volume of 0.1 N NaOH in mL; 9.94 is the theoretical value of % NH_2_ group content of chitosan (the molecular weight of glucosamine unit is 161 and molecular weight of amino group is 16, hence, amino group content in percentage is 9.94); and 16 is the gram equivalent weight of the amino group i.e. –NH_2_. Notably, in this equation, the concentration terms of aqueous standardized HCl and NaOH solutions in normality are deliberately omitted to increase the ease of calculation. Moreover, to use the above-mentioned equation, 0.1 N HCl and 0.1 N NaOH should always be prepared to carry out acid-base titration for the estimation of DD value of chitosan.

Moreover, it is customary to take precautions such as carefully noting burette’s readings, not allowing air gap in the burette, etc. while titrating, which will help provide an accurate result and enable minimizing experimental errors too.

### 2.4 Characterizations of chitosan samples

#### 2.4.1 Fourier transform infrared spectroscopy (FT-IR)

The FT-IR spectra of CS1, CS2, and CS3 samples were recorded using Bruker Tensor 37 spectrometer. The spectra were recorded in spectral range from 4000 cm^−1^ to 500 cm^−1^. Various absorption band ratios and equations have been used by many researchers to compute degree of deacetylation of chitosan using the FT-IR spectroscopy [5, 21, 22, 23, 24, 25]. Almost none of them does not give satisfactory results. However, after a thorough reviewing, we used Eq. (2) as reported by Brugnerotto *et al.* [26] to calculate degree of acetylation (DA) of chitosan samples with the help of the absorption bands at 1420 cm^−1^ and 1320 cm^−1^ followed by subtracting the DA of each chitosan from 100 for obtaining DD value of each chitosan as shown in Eq. (3):

(2) A_1320_/A_1420_ = 0.3822 − 0.03133 [26].

(3) DD (%) = 100 − DA (%) [26].

#### 2.4.2 Nuclear magnetic resonance

The solid-state NMR experiments were performed on ASCEND™ 400 Bruker, using a Bruker solid state probe, operating at 100 MHz and 512 scans for ^13^C. The powdered samples were packed into 2.5 mm zirconia rotors and all the spectra were recorded at 25 °C. To find out the degree of acetylation (DA) of chitosan, the integral of the methyl carbon was divided by 1/6th of the summation of integrals of carbon atoms from the d-glucopyranosyl ring (C1–C6 atoms) followed by multiplying the whole by 100 as shown in Eq. (4) [27]. Similarly, Eq. (3) was used to determine the DD of chitosan.

(4) $$DA=\frac{\int N-CH3}{\frac{1}{6}\left(\int C1+\int C2+\int C3+\int C4+\int C5+\int C6\right)}\times 100$$

where, ∫N–CH_3_ is the integral of methyl carbon, and ∫_C1_ + ∫_C2_ + ∫_C3_ + ∫_C4_ + ∫_C5_ + ∫_C6_ is the summation of integrals of carbon atoms of the d-glucopyranosyl ring.

### 2.5 Statistical analysis

All experimental values are portrayed as mean ± S.D. The data were analyzed using one-way ANOVA. Moreover, Tukey HSD test and one sample T test were also performed to analyze multiple comparisons between the groups of CS samples and within the groups of each chitosan sample respectively by using p-value at less than 0.05 to ascertain whether the values obtained through experimentation are significantly different.

## 3 Results and discussion

### 3.1 Acid-base titration

In this study, the acid-base titration method was successfully used for the determination of the degree of deacetylation of various grades of chitosan. Undoubtedly, the said method is very cost-effective but sample preparation takes longer time for performing this titration. In contrast to the traditional method, chitosan is initially dissolved in HCl for converting its –NH_2_ group into its corresponding form of –NH_3_^+^ (protonated amino group), which facilitates developing acidic character into chitosan solution and then it is titrated against standard NaOH in presence of methyl orange as a pH indicator, which helps us understand the neutralization point of the reaction. The reactions involved while carrying out this titration are shown below.

Before titration,

**Figure:**
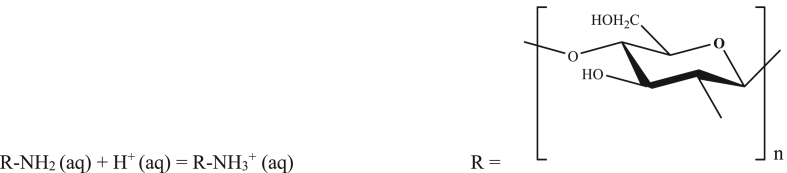

During titration.

Step 1:

H^+^ (aq) + OH¯ (aq) = H_2_O (l)

Step 2:

R-NH_3_^+^ (aq) + OH¯ (aq) = R-NH_2_ (aq) + H_2_O (l)

**Figure 2:**
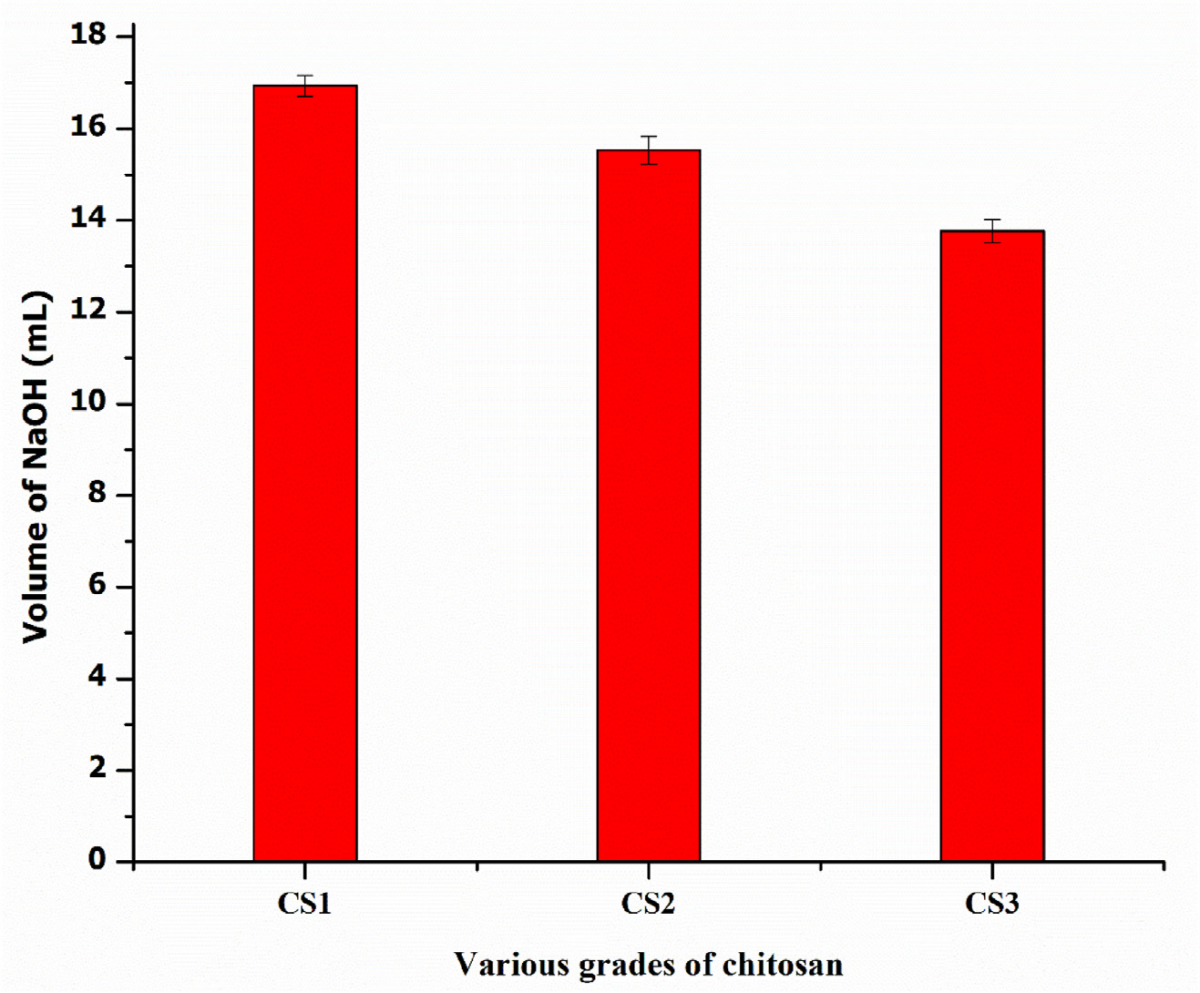
Consumption of NaOH for each 30 mL of various grades of chitosan during acid-base titration.

From the above results, it can be predicted that the excess amount of HCl in test chitosan solution is neutralized first by OH¯ ions before starting the second step reaction during titration. In the second step, the protonated amino groups were converted to corresponding amino groups and colour change was observed from pink to yellow orange, which indicated the end point. Finally, the exact volume of NaOH required to neutralize the individual chitosan solution was noted down to separately determine DD for our three different grades of chitosan using the equation as mentioned above. Notably, the moisture content of the test chitosan samples was in the range of 9–10%. Further, a plot between volume of NaOH and various grades of chitosan is shown in Figure 2.

Moreover, from the data presented in Table 1, it is clearly seen that for various grades of chitosan with varying degree of deacetylation, the consumption of NaOH is different for each of them. Therefore, it can be said that the higher the mean volume of NaOH the lower is the DD value of chitosan and vice-versa.

**Table 1:** Variation of consumption of NaOH during acid-base titration with DD value of chitosan.

Sample	Volume of chitosan solution against each titration (mL)	Volume of NaOH (Mean ± SD)	Experimental DD value (%)	DD value provided by manufacturer
CS1	30	16.93 ± 0.23	77.04 ± 1.36	78.26
CS2		15.53 ± 0.31	81.71 ± 1.73	82.30
CS3		13.77 ± 0.25	91.68 ± 1.42	86.18

It is quite desirable because percentage of amino group content is more in case of higher deacetylated chitosan, which in turn, exhibits less acidic character and thus, results in the requirement of lesser amount of NaOH compared to low deacetylated chitosan during acid-base titration. In other words, due to more numbers of amino groups for higher deacetylated chitosan, it will attract more numbers of hydrogen ions (protons), which in turn, will decrease the concentration of hydrogen ions in the chitosan solution, thus resulting in the consumption of lesser amount of NaOH. The mean volume of NaOH consumed against each chitosan sample was used to estimate DD of various grades of chitosan and their corresponding experimental DD values were compared with the ones supplied by the manufacturer. It was found that the respective experimental DD values of CS1 and CS2, and CS3 were 77.04% and 81.71% whereas the same for the aforesaid samples were 78.26% and 82.30% respectively as provided by the manufacturer. From the results, it can be said that the calculated DD values using our established equation are almost same with the values of chitosan samples supplied by the manufacturer but in case of CS3, the percentage DD value (91.68) is little higher than the expected value i.e., 86.18 and it is assumed that it may be due to experimental errors occurring at the time of handling. Further, a statistical analysis (data not shown) was performed to check whether the values displayed in Table 1 were significantly different. From Tukey HSD test at the 0.05 level, it was found that all the mean volumes of NaOH for each chitosan sample were significantly different from each other. On the other hand, by performing one sample T test, it was found that all the respective volumes of NaOH against each chitosan sample were not significantly different and which is extremely desirable. From these results, it was clear that all the measurements during titration were recorded fantastically.

Apart from using our proposed equation, other equations reported by other researchers had also been used for the estimation of DD of our chitosan samples for comparison. None of them provided any fruitful results. Moreover, Yao *et al.* [17] have shown the inappropriateness of using the method based on Chinese pharmacopoeia and the European pharmacopoeia for the estimation of degree of deacetylation of chitosan using methyl orange as an indicator because of distinctly and accurately recognizing the colour change from red to yellow at the completion of the reaction. Therefore, we avoided using this method but used methyl orange as an indicator for our study and finally we got an accurate result using the acid-base titration as discussed in the foregoing section. In another study, Hossain and Uddin [18] also demonstrated the use of the same method using methyl orange as a pH indicator except heating chitosan solution at 60 °C while titrating against HCl. In another attempt, Varan [19] reported the same acid-base titration method using phenolphthalein as an indicator. Similarly, Youling *et al.* [20] also demonstrated on the determination of DD value of chitosan using acid-base titration method. However, only the basic difference is that all of them used different equations to obtain DD value for their respective samples using the same titration with little modification. Notably, as shown in Table 2, for cross-verification once we used various equations to solve our own problem, different results were obtained rather we got very absurd results, which were really a matter of concern. This drastic variation in terms of results greatly demands to introduce a new and simplified equation. Hence, it is expected that our newly introduced equation for the estimation of DD of chitosan would remove all the confusions over the already mentioned equations and help researchers to deploy this simplified method along with the equation to estimate DD of any chitosan sample in an accurate manner.

**Table 2:** Estimation of degree of deacetylation of our chitosan samples via acid-base titration using various equations as reported by various researchers.

Sr. No.	Equation	Degree of deacetylation (DD)			Reference
		CS1	CS2	CS3	
1.	$NH2\%=\frac{\left[\left(C1V1-C2V2\right)\times 0.016\right]}{G\left(100-W\right)}\times 100$ $DD\left(\%\right)=\frac{\%\;of\;NH2}{9.94}\times 100$	0.231	0.245	0.27	[1]
2.	$DD\%=\frac{\left(CHCl\times VHCl-CNaOH\;\times VNaOH\right)\times 0.016\%\times 100\%}{G\times \left(100-W\right)\times 9.94\%}$	0.002	0.002	0.003	[17]
3.	$DD\%=\frac{\left(C1\times V1-C2\;\times V2\right)\;\times 0.016}{M\;\times 0.0994}$	0.232	0.245	0.275	[18]
4.	$DD\%=\left\{\frac{\left[\left(V2-V1\right)\;\left(L\right)\times MNaOH\;\left(\frac{mol}{L}\right)\times Unit\;mass\;of\;chitosan\;\left(\frac{g}{mol}\right)\right]}{Mass\;of\;chitosan\;sample\;\left(\frac{g}{mol}\right)}\right\}\times 100$	0.144	0.154	0.171	[19]

Nevertheless, we got nearly the same results every time by using the acid-base titration and the proposed equation as well. It not only clearly indicated the reproducibility, accuracy, and reliability of the method adopted by the authors but also boosted up the efficiency of the newly established equation in terms of determining the DD value of our chitosan as already shown in Table 1. Moreover, the method adopted here is much more convenient over the other existing acid-base titration methods in terms of its simplicity and reproducibility to estimate the degree of deacetylation of chitosan. Notably, the reproducibility had been checked at least 3–4 times by conducting acid-base titration using these three test chitosan samples followed by validation with ^13^C-NMR technique to be discussed in section 3.3.

### 3.2 FT-IR

**Figure 3:**
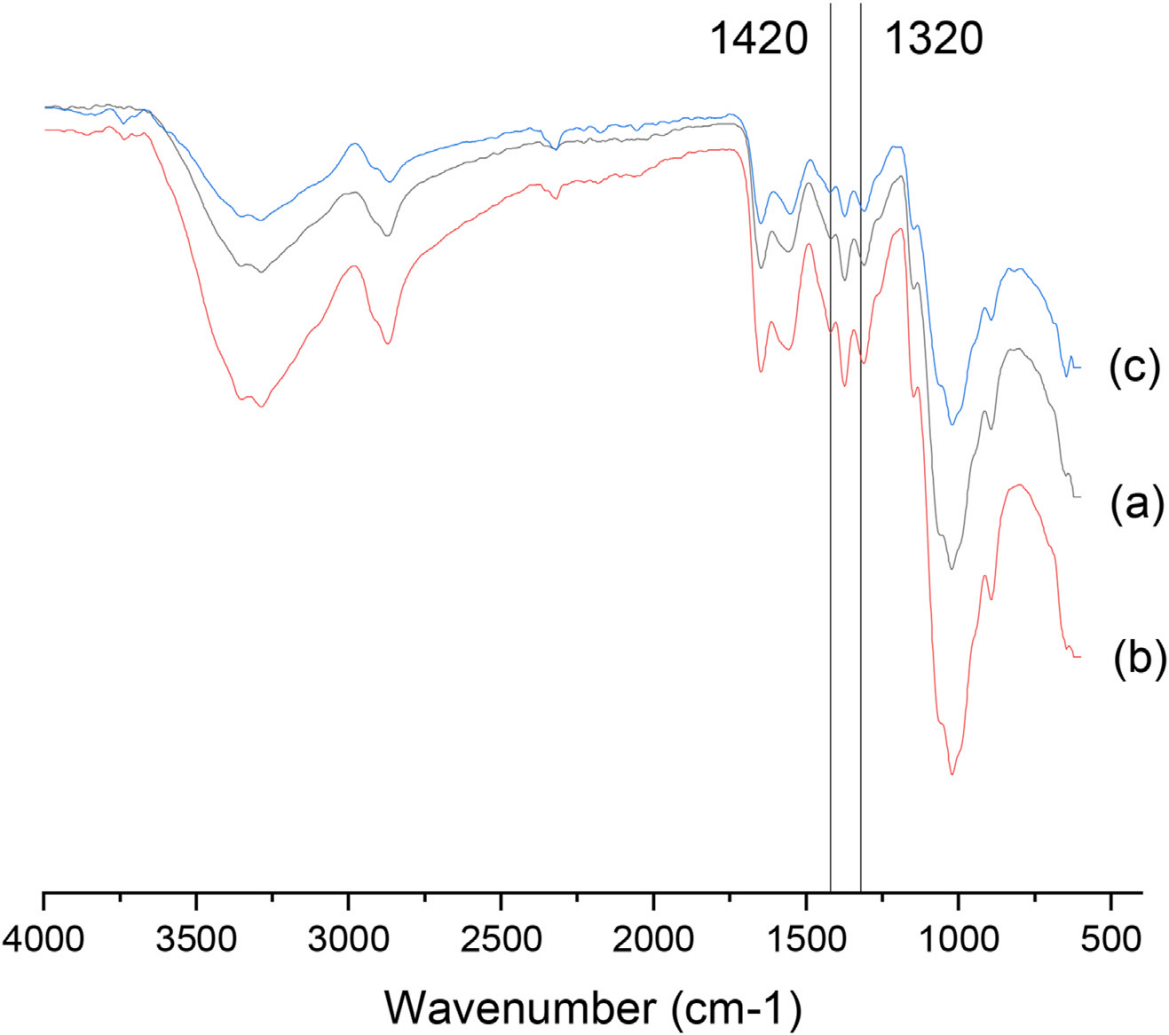
Estimation of degree of deacetylation from the individual absorption bands at 1420 cm^−1^ and 1320 cm^−1^ of FT-IR spectra of (a) CS1, (b) CS2, and (c) CS3.

FT-IR spectroscopy was employed to estimate DD values of different grades of chitosan samples using Eq. (3) as proposed by Brugnerotto *et al.* [26] from the ratio of the absorbance of the amide III at 1320 cm^−1^ to the absorbance of the CH_3_ bending at 1420 cm^−1^ in the individual spectrum as depicted in Figure 3. The calculated DD values for our test chitosan samples, namely CS1, CS2, and CS3 were 76.09%, 78.04%, and 76.22% respectively.

The calculated DD value of only CS1 using FT-IR spectroscopy was as nearly the same as the value obtained by acid-base titration i.e., 77.04%. Moreover, it also approximately satisfied the value i.e., 78.26% as supplied by the chitosan manufacturer. On the other hand, the calculated DD values for the remaining two chitosan samples were inconsistent with the experimental ones using acid-base titration as well as the same reported by the manufacturer. In addition, various baselines were also studied to calculate the degree of deacetylation of chitosan (data not shown) but none of them gave accurate results. This similar kind of phenomenon was also reported by Shigemasa *et al.* [24], while evaluating the various absorbance ratios from infrared spectral data for estimating the degree of deacetylation of chitin. It was further corroborated with the fact that the use of various baselines in FT-IR spectra of chitosan would inevitably contribute to variations in DD values [23, 25, 28]. In addition, some other factors including sample preparation, type of instrument, and conditions may also greatly influence the sample analysis in terms of determining DD of chitosan [25, 28, 29].

### 3.3 ^13^C-NMR

**Figure 4:**
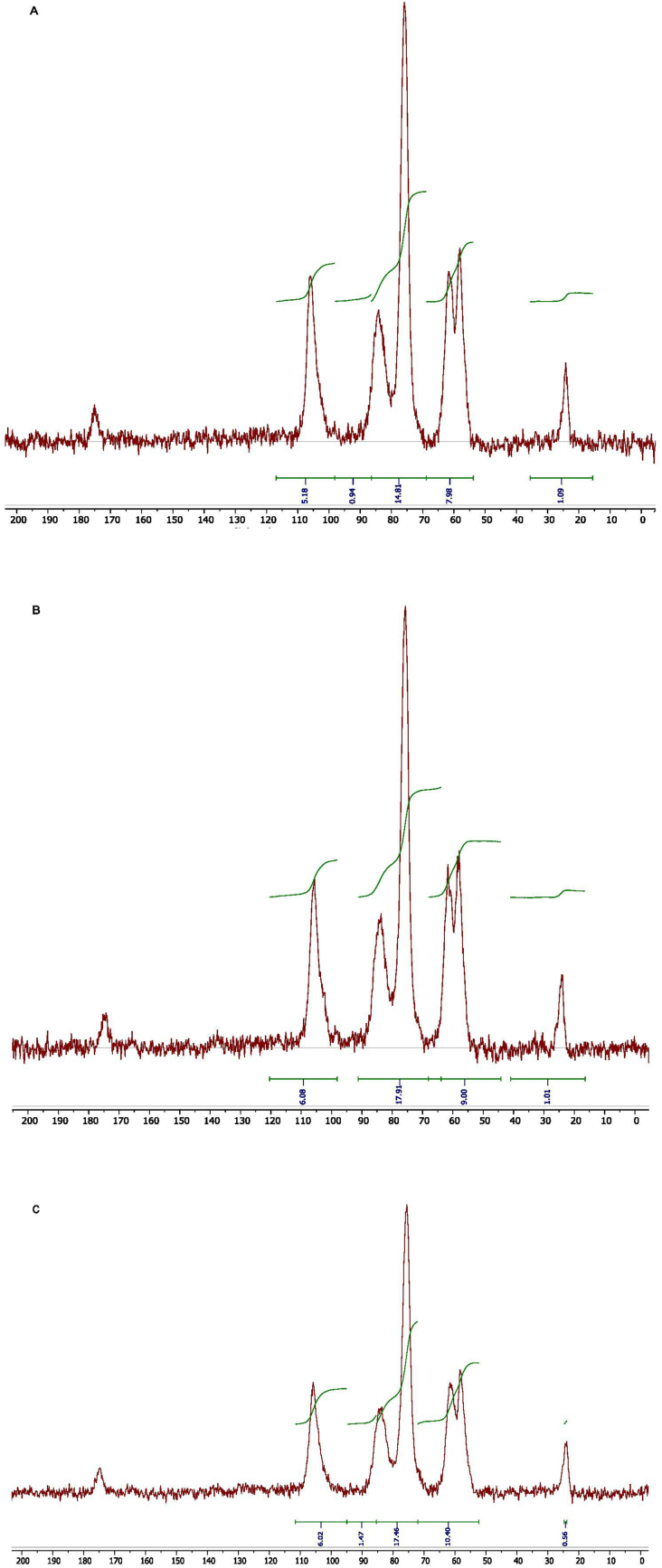
^13^C/CP/MAS NMR spectra of (A) CS1, (B) CS2 and (C) CS3 for the estimation of their deacetylated values.

Considering the flaws as mentioned in the section of FT-IR, ^13^C-NMR spectroscopy was employed to validate the DD values of our chitosan samples as obtained by the acid-base titration. Notably, it is quite evident that ^13^C-NMR in solid state is very useful not only for analysing the structure of chitosan but can also be deployed to determine its degree of deacetylation [13, 27, 30, 31]. Chemical shifts of various grades of chitosan are shown in Table 3. As can be seen in Figure 4, the peaks of C3 and C5 in each NMR spectrum are completely overlapped with each other and appeared as a single signal around at δ 76 ppm. Further, all the corresponding peaks of all the chitosan test samples at various carbon atoms obtained were validated with the ^13^C-NMR spectrum of a commercial chitosan sample, which was already reported in the literature [26].

**Table 3:** Chemical shifts of our chitosan samples obtained by ^13^C/CP/MAS NMR.

	CS1	CS2	CS3
C=O	175.23	174.13	174.79
C1	106.16	105.73	105.8
C4	84.09	83.73	84.09
C3 or C5	75.84	75.69	75.54
C6	61.58	61.66	61.59
C2	58.06	57.93	58.39
CH_3_	24.17	24.02	24.02

The respective DD values of CS1, CS2, and CS3 calculated from their corresponding peak areas were 77.39%, 81.64%, and 90.50%. Further, all these DD values were in good agreement with the ones obtained by the acid-base titration method. Moreover, all these DD values were also perfectly in line with the ones reported by the manufacturer. On the other hand, the trend of decreasing peak area under the peak at δ 24 ppm as shown in Figure 4 (A-C) also reveals the tendency of increasing degree of deacetylation as reported elsewhere [22]. Thus, it is a clear indication of the authenticity of the adopted acid-base titration method [1] with a new approach as well as the newly established equation reported here to determine the degree of deacetylation of chitosan. All the DD values obtained by various methods are comprehensibly summarized in Table 4.

**Table 4:** Estimation of degree of deacetylation (DD) values of various chitosan samples using different methods.

Samples	DD values in percentage		
	Acid-base titration Mean ± SD	FT-IR Spectroscopy	NMR Spectroscopy
CS1	77.04 ± 1.36 (78.26)	76.90	77.39
CS2	81.71 ± 1.73 (82.30)	78.04	81.64
CS3	91.68 ± 1.42 (86.18)	76.22	90.50

∗DD values mentioned in the parentheses are provided by manufacturer.

## 4 Conclusion

The acid-base titration method reported by Sivashankari *et al.* [1] was used for the determination of DD for various grades of chitosan procured from the Marine Hydrocolloids, Kochi, Kerala, India. The experimental DD values estimated using the newly established equation through the acid-base titration method were 77.04%, 81.71%, and 91.68% for CS1, CS2, and CS3 respectively. All these values were consistent with the DD values mentioned in the specification of the said chitosan samples. Moreover, ^13^C-NMR spectroscopy was also employed to validate the data obtained by the acid-based titration method. The respective DD values calculated from NMR data for CS1, CS2, and CS3 were 77.39%, 81.64%, and 90.50%, which in turn, were in good agreement with the same obtained by the acid-base titration method. On the other hand, almost all the DD values were inconsistent with the ones calculated from FT-IR spectra of various grades of chitosan. So always we cannot rely on DD values of chitosan obtained by using FT-IR spectroscopy rather we can undoubtedly rely on NMR spectroscopy because of its extremely sensitivity and high precision. Thus, it is suggested that the researchers, manufacturers, etc. should mention the method while reporting DD values of chitosan. However, apart from using other techniques, the acid-base titration method along with the newly established equation can be used for the estimation of DD values of chitosan. It is worth mentioning that the acid-base titration method is an inaccurate method not only because of human error but also because of the inappropriate equations reported so far in the literature. If the methodology be carefully adopted as described in the experimental section and the newly established equation be used then every time, there are chances of getting nearly same results and thus, it would be more beneficial, which could obviate the need of using expensive techniques, such as NMR for the estimation of DD value of chitosan. That is what is the novelty of this work. Therefore, it can be concluded that the acid-base titration method with a new approach along with the established equation mentioned here can flawlessly be used to determine DD value of any chitosan sample of interest for further research in terms of its relevant applications.

## Declarations

### Author contribution statement

Joydeep Dutta: Conceived and designed the experiments; Analyzed and interpreted the data; Contributed reagents, materials, analysis tools or data; Wrote the paper.

Priyanka: Performed the experiments; Contributed reagents, materials, analysis tools or data; Wrote the paper.

### Funding statement

This research did not receive any specific grant from funding agencies in the public, commercial, or not-for-profit sectors.

### Data availability statement

The data that has been used is confidential.

### Declaration of interests statement

The authors declare no conflict of interest.

### Additional information

No additional information is available for this paper.

## References

[1] Sivashankari P.R., Prabaharan M., Jennings J.A., Bumgardner J.D. (2017). Chitosan Based Biomaterials.

[2] Murali S., Kumar S., Koh J., Seena S., Singh P., Ramalho A., Sobral A.J.F.N. (2019). Bio-based chitosan/gelatin/Ag@ZnO bionanocomposites: synthesis and mechanical and antibacterial properties. Cellulose.

[3] Li P., Zhao J., Chen Y., Cheng B., Yu Z., Zhao Y., Yan X., Tong Z., Jin S. (2017). Preparation and characterization of chitosan physical hydrogels with enhanced mechanical and antibacterial properties. Carbohydr. Polymer.

[4] Takarina N.D., Nasrul A.A., Nurmarina A. (2017). Degree of deacetylation of chitosan extracted from white Snapper (Lates sp.) Scales waste. Int. J. Pharma Med. Biol. Sci..

[5] Khan T.A., Peh K.K., Ch’ng H.S. (2002). Reporting degree of deacetylation values of chitosan: the influence of analytical methods. J. Pharm. Pharmaceut. Sci..

[6] Renata C.-B., Diana J., Bozena R., Piotr U., Rosiak J.M. (2012). Determination of degree of deacetylation of chitosan - comparision of methods. Prog. Chem. Appl. Chitin its Deriv..

[7] Kumari S., Rath P., Sri Hari Kumar A., Tiwari T.N. (2015). Extraction and characterization of chitin and chitosan from fishery waste by chemical method. Environ. Technol. Innovat..

[8] Chou S.-F., Lai J.-Y., Cho C.-H., Lee C.-H. (2016). Relationships between surface roughness/stiffness of chitosan coatings and fabrication of corneal keratocyte spheroids: effect of degree of deacetylation. Colloids Surf. B Biointerfaces.

[9] Luo L.J., Huang C.C., Chen H.C., Lai J.Y., Matsusaki M. (2018). Effect of deacetylation degree on controlled pilocarpine release from injectable chitosan-g-poly(N-isopropylacrylamide) carriers. Carbohydr. Polym..

[10] Yan N., Wan X.F., Chai X.S. (2019). Rapid determination of degree of deacetylation of chitosan by a headspace analysis based Titrimetric technique. Polym. Test..

[11] Matica M.A., Aachmann F.L., Tøndervik A., Sletta H., Ostafe V. (2019). Chitosan as a wound dressing starting material: antimicrobial properties and mode of action. Int. J. Mol. Sci..

[12] Kumirska J., Weinhold M.X., Thöming J., Stepnowski P. (2011). Biomedical activity of chitin/chitosan based materials- influence of physicochemical properties apart from molecular weight and degree of N-Acetylation. Polymers (Basel).

[13] Kasaai M.R. (2010). Determination of the degree of N-acetylation for chitin and chitosan by various NMR spectroscopy techniques: a review. Carbohydr. Polym..

[14] Weißpflog J., Vehlow D., Müller M., Kohn B., Scheler U., Boye S., Schwarz S. (2021). Characterization of chitosan with different degree of deacetylation and equal viscosity in dissolved and solid state – insights by various complimentary methods. Int. J. Biol. Macromol..

[15] dos Santos Z.M., Caroni A.L.P.F., Pereira M.R., da Silva D.R., Fonseca J.L.C. (2009). Determination of deacetylation degree of chitosan: a comparison between conductometric titration and CHN elemental analysis. Carbohydr. Res..

[16] Zhang Y., Zhang X., Ding R., Zhang J., Liu J. (2011). Determination of the degree of deacetylation of chitosan by potentiometric titration preceded by enzymatic pretreatment. Carbohydr. Polym..

[17] Jiang Y., Fu C., Wu S., Liu G., Guo J., Su Z. (2017). Determination of the deacetylation degree of chitooligosaccharides. Mar. Drugs.

[18] Hossain S., Uddin M.K. (2020). Isolation and extraction of chitosan from shrimp shells. Int. J. Adv. Res..

[19] Varan N. (2017). The use of titration technique and FTIR bands to determine the deacetylation degree of chitosan samples. J. Textil. Sci. Eng..

[20] Youling Y., Chesnutt B.M., Haggard W.O., Bumgardner J.D. (2011). Deacetylation of chitosan: material characterization and in vitro evaluation via albumin adsorption and pre-osteoblastic cell cultures. Materials (Basel).

[21] Zhang Y., Xue C., Xue Y., Gao R., Zhang X. (2005). Determination of the degree of deacetylation of chitin and chitosan by X-ray powder diffraction. Carbohydr. Res..

[22] Lertwattanaseri T., Ichikawa N., Mizoguchi T., Tanaka Y., Chirachanchai S. (2009). Microwave technique for efficient deacetylation of chitin nanowhiskers to a chitosan nanoscaffold. Carbohydr. Res..

[23] Baxter A., Dillon M., Taylor K.D.A., Roberts G.A.F. (1992). Improved method for i.r. determination of the degree of N-acetylation of chitosan. Int. J. Biol. Macromol..

[24] Shigemasa Y., Matsuura H., Sashiwa H., Saimoto H. (1996). Evaluation of different absorbance ratios from infrared spectroscopy for analyzing the degree of deacetylation in chitin. Int. J. Biol. Macromol..

[25] Knidri H.E., Khalfaouy R.E., Laajeb A., Addaou A., Lahsini A. (2016). Eco-friendly extraction and characterization of chitin and chitosan from the shrimp shell waste via microwave irradiation. Process Saf. Environ. Protect..

[26] Desbrie J., Brugnerotto J., Lizardi J., Goycoolea F.M., Argu W. (2001). An infrared investigation in relation with chitin and chitosan characterization. Polymer.

[27] Song C., Yu H., Zhang M., Yang Y., Zhang G. (2013). Physicochemical properties and antioxidant activity of chitosan from the blowfly Chrysomya megacephala larvae. Int. J. Biol. Macromol..

[28] Sannan T., Kurita K., Ogura K., Iwakura Y. (1978). Studies on chitin: 7. I.r. spectroscopic determination of degree of deacetylation. Polymer.

[29] Sabnis S., Block L.H. (1997). Improved infrared spectroscopic method for the analysis of degree of N-deacetylation of chitosan. Polym. Bull..

[30] Duarte M.L., Ferreira M.C., Marvao M.R., Rocha Joao (2001). Determination of the degree of acetylation of chitin materials by 13C CP/MAS NMR spectroscopy. Int. J. Biol. Macromol..

[31] Heux L., Brugnerotto J., Desbrieres J., Versali M.-F., Rinaudo M. (2000). Solid state NMR for determination of degree of acetylation of chitin and chitosan. Biomacromolecules.

